# Decreased vascular contractility induced by hemin is associated with a reduced rho-kinase activity

**DOI:** 10.5830/CVJA-2013-005

**Published:** 2013-04

**Authors:** Bonaventure Awede, Marie-Christine Lemaire, Pierre Bonnet, Veronique Eder

**Affiliations:** LAB.P.ART.-EA3852, Faculty of Medicine, University of Tours, 10 bis Boulevard Tonnellé, Tours, France; Unité de Physiologie, Faculté des Sciences de la Santé, Université d’Abomey-Calavi, Bénin; LAB.P.ART.-EA3852, Faculty of Medicine, University of Tours, 10 bis Boulevard Tonnellé, Tours, France; LAB.P.ART.-EA3852, Faculty of Medicine, University of Tours, 10 bis Boulevard Tonnellé, Tours, France; LAB.P.ART.-EA3852, Faculty of Medicine, University of Tours, 10 bis Boulevard Tonnellé, Tours, France

**Keywords:** haeme oxygenase, vascular contractility, rho-kinase

## Abstract

**Objectives:**

In this study, the role of rho-kinase activity in the modulation of vascular contractility induced by hemin, a heme oxygenase inducer, was investigated.

**Methods:**

Aortic rings from Wistar rats were incubated in physiological saline solution (PSS) containing hemin at 10-4 M for six hours then contracted with phenylephrine, and a dose-response curve was established. The effect of Y-27632, a rho-kinase inhibitor, on the relaxation of the pre-contracted aortic rings was then studied.

**Results:**

Incubation of the aortic rings in hemin induced an increased expression of heme oxygenase 1 (HO-1). A reduction in the contractile force of aortic rings incubated in hemin was observed in response to phenylephrine. Y-27632 at a concentration of 10-6 M induced a 36% relaxation of the control aortic rings but only a 20% relaxation in aortic rings treated with hemin.

**Conclusion:**

These data suggest that the decreased vascular contractility induced by hemin could, in part, result from an inhibition of rho-kinase activity.

## Abstract

Carbon monoxide (CO), like nitric oxide (NO), has been shown to decrease vascular contractility. This gas is endogenously produced by the breakdown of heme into biliverdin, iron and CO, and the reaction is catalysed by heme oxygenase. Heme oxygenase (HO) exists in three isoforms: an inductive form, HO-1, and two constitutive forms, HO-2 and HO-3.^1^ Hemin is one of the components that induces expression of HO-1 in vascular tissues both *in vitro* and *in vivo*. Hemin is also a substrate of heme oxygenase.

*In vitro* induction of HO-1, which results in CO production, decreased both animal and human arterial contractility.^2^,^3^ Longterm *in vivo* administration of hemin to spontaneous hypertensive rats (SHR) has been shown to normalise arterial pressure.^4^

If smooth muscle contraction is regulated by the cytosolic calcium concentration, which induces activation of myosin light-chain kinase and then phosphorylation of the regulatory myosin light chain, it is also regulated by the calcium sensitivity of myofilaments. This mechanism is partly achieved by the inhibition of myosin light-chain phosphatase, and the small GTPase rho and its target rho-associated kinase participate in this inhibition.^5^,^6^ Increased activity of rho-kinase has been observed in many models of arterial hypertension, and administration of inhibitors of rho-kinase activity has been shown to lower blood pressure.^7^-^10^ Activation of rho-kinase is also involved in many other cardiovascular disorders.^11^-^13^

Previous studies have shown that the effects of carbon monoxide or heme oxygenase 1 induction on vascular contractility or blood pressure occur via activation of soluble guanylate cyclase, which results in the production of cyclic GMP and via activation of the potassium current.^3^,^4^,^14^-^16^ The possible involvement of rho-kinase has never been investigated *in vitro*.

In this study, we hypothetised that induction of heme oxygenase could result in decreased activation of rho-kinase. Therefore the effect of inhibition of rho-kinase on the relaxation of pre-contracted aortic rings was investigated following incubation in hemin.

## Methods

All animal experimental protocols were performed with the approval of the regional ethics committee (CREEA no CL2007-013). Twelve-week-old rats used in this study were housed at 21°C with 12-hour light/dark cycles. They were fed with standard laboratory food and had unlimited access to drinking water. Hemin solution was prepared by dissolving hemin (Fluka, France) in a phosphate buffer solution (PBS) pH 12 and adjusted to pH 7.4. Animals were anesthetised by an intraperitoneal injection of sodium pentobarbital (100 mg/kg of body weight).

## Contraction of isolated artery rings

The rats were euthanised and the thoracic aorta was dissected. Transverse ring sections were isolated and the endothelium was destroyed by rubbing the intimal surface with forceps. The rings were then suspended between two wires connected to the bottom of an organ bath and to an isometric force transducer that allowed the recording of the tension developed by each ring.

In all experiments, the rings were stretched against a 3-g pre-load in the organ bath, which was filled with physiological saline solution (PSS) at 37°C, containing (in mM): NaCl 138.6; KCl 5.4; CaCl_2_ 1.8; MgCl_2_ 1.2; NaH_2_PO_4_ 0.33; HEPES 10 and glucose 11. The pH was adjusted to 7.4 using NaOH.

After one hour of rest (equilibrium), the rings were pre-contracted with a PSS solution containing 80 mM K^+^ (K80) in order to provide maximal contractile amplitude of tone. This was used as a reference and to check tissue viability. A relaxation response to acetylcholine was used to confirm the absence of endothelium.

The vascular rings were then contracted with a cumulative dose of phenylephrine (10^-9^ to 10^-6^ M). After washing the rings to suppress the effects of phenylephrine, the rings were incubated in PSS containing hemin at a concentration of 10^-4^ M. After six hours of incubation, the rings were contracted again with a cumulative dose of phenylephrine.

## Relaxation effect of Y-27632

Relaxation induced by Y-27632 [(R)-(+)-trans-N-(4-pyridyl)-4-(1-aminoethyl)-cyclohexanecarboxamide] (Tocris, France), a rho-kinase inhibitor, was tested on either the control rings or rings incubated in hemin following contraction induced by phenylephrine at 10^-6^ M. Y-27632 was used at a concentration of 3 × 10^-7^ M. Results were expressed as a percentage of the magnitude of contraction with 10^-6^ M of phenylephrine.

All the data were collected with a computerised data-acquisition system using Genie software (Adventech, USA). All the analyses were carried out using Origin 6 software (Microcal Software, Northampton, MA, USA).

## Immunohistochemistry

Aortic rings incubated in either PSS or PSS with hemin at 10^-4^ M were embedded in ‘optimal cutting temperature compound’ (Tissue-Tek^R^ OCT compound, Sakura Finetek, France) and then frozen in liquid nitrogen. Transverse sections of 7 μm were made using a cryostat and mounted on a microscope slide. Immunohistochemical analysis of HO-1 was performed.

Sections were first incubated with a rabbit polyclonal anti-rat HO-1 antibody at room temperature for four hours. The antigen–antibody reaction was detected using a molecular probe Alexa fluor dye goat anti-rabbit secondary antibody (Interchim, France). The positive reaction was visualised under confocal microscopy.

## Statistical analysis

Results are expressed as means ± SEM. Differences between control and hemin groups were compared using Student’s *t*-test and the level of statistical significance was set at *p* < 0.05.

## Results

**Fig. 1.**
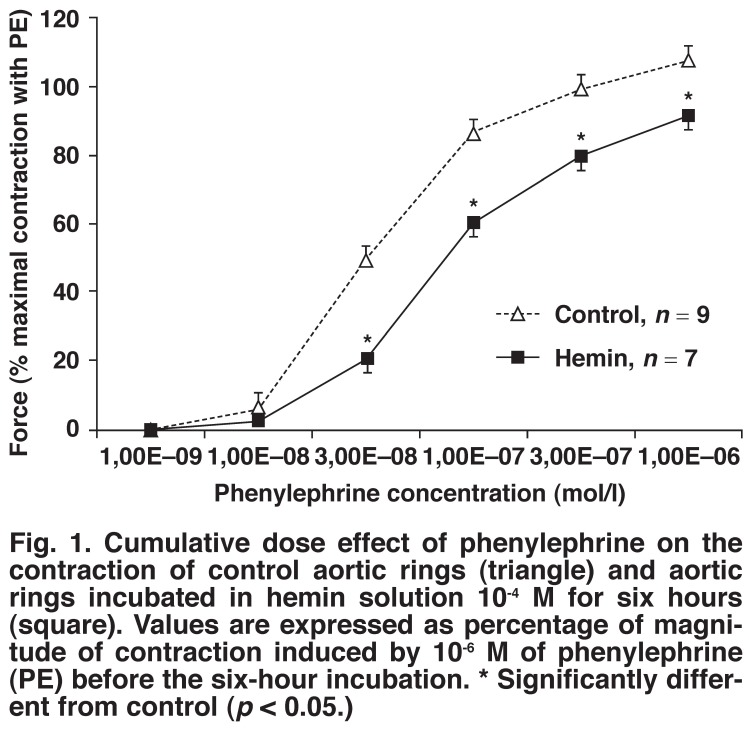
Cumulative dose effect of phenylephrine on the contraction of control aortic rings (triangle) and aortic rings incubated in hemin solution 10^-4^ M for six hours (square). Values are expressed as percentage of magnitude of contraction induced by 10^-6^ M of phenylephrine (PE) before the six-hour incubation. * Significantly different from control (*p* < 0.05.)

Cumulative concentrations of phenylephrine induced a concentration-dependent increase in the contraction of aortic rings. As shown in Fig. 1, incubation of aortic rings in hemin solution induced a decrease of the contractile force of the aortic rings at all concentrations of phenylephrine from 3 × 10^-8^ to 10^-6^ M.

**Fig. 2.**
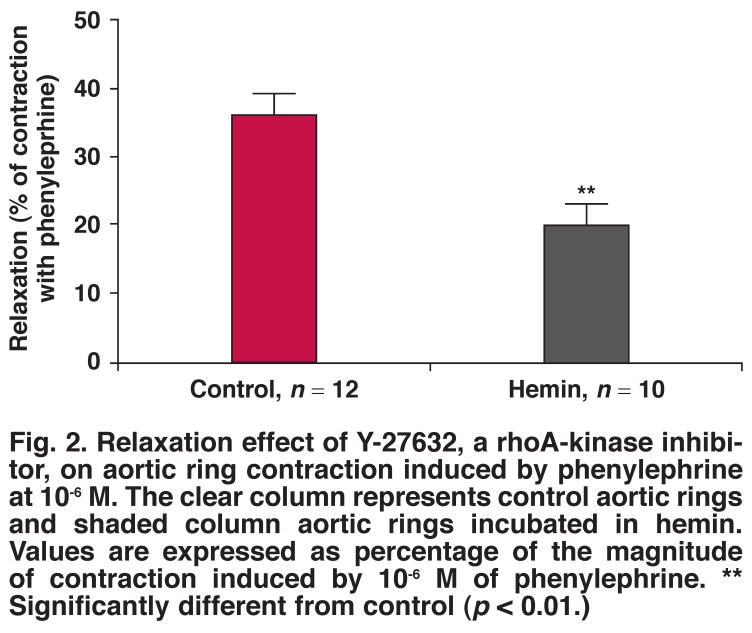
Relaxation effect of Y-27632, a rhoA-kinase inhibitor, on aortic ring contraction induced by phenylephrine at 10^-6^ M. The clear column represents control aortic rings and shaded column aortic rings incubated in hemin. Values are expressed as percentage of the magnitude of contraction induced by 10^-6^ M of phenylephrine. ** Significantly different from control (*p* < 0.01.)

The application of Y-27632, a specific and potent rho-kinase inhibitor, induced a relaxation in the isolated aortic rings. In the control aortic rings, the magnitude of the relaxation at 3 × 10^-7^ M of Y-27632 was 36% of the contraction induced by 10^-6^ M of phenylephrine. In aortic rings treated with hemin, the relaxation induced by Y-27632 was reduced to 20% of the contraction induced by phenylephrine (Fig. 2).

**Fig. 3.**
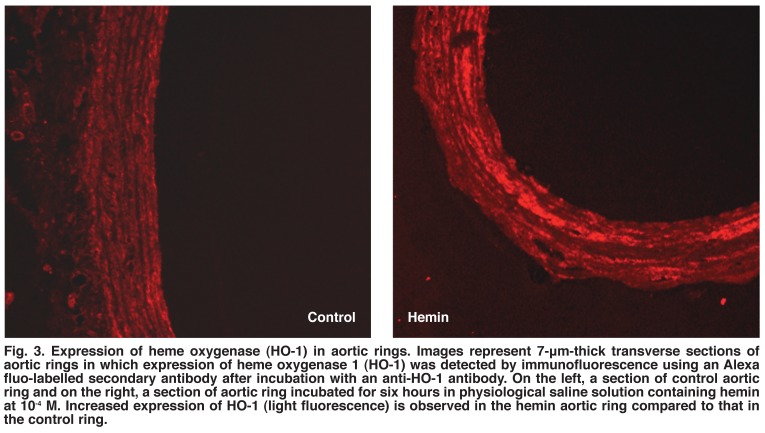
Expression of heme oxygenase (HO-1) in aortic rings. Images represent 7-μm-thick transverse sections of aortic rings in which expression of heme oxygenase 1 (HO-1) was detected by immunofluorescence using an Alexa fluo-labelled secondary antibody after incubation with an anti-HO-1 antibody. On the left, a section of control aortic ring and on the right, a section of aortic ring incubated for six hours in physiological saline solution containing hemin at 10^-4^ M. Increased expression of HO-1 (light fluorescence) is observed in the hemin aortic ring compared to that in the control ring.

Immunohistochemical study showed expression of HO-1 in both control and hemin-treated aortic rings. As shown in Fig. 3, six-hour incubation of aortic rings in hemin resulted in an increased expression of HO-1.

## Discussion

The main findings of this study were that the decreased contractility of the aortic rings induced by hemin was associated with a reduced effect of Y-27632, a rho-kinase inhibitor, on the relaxation of aortic rings pre-contracted with phenylephrine. The reduced vascular smooth muscle contractile force induced by hemin found in this study was associated with an increased expression of heme oxygenase HO-1. This is in agreement with data previously published on rat and human vessels.^3^

The change in contractile force produced was observed after six hours of incubation in hemin, not after four hours, as was observed in human vessels. The four-hour duration in our study was without significant effect on the phenylephrine-induced contraction of aortic rings.

The hemin effect on vascular contractility occurs via mechanisms involving heme oxygenase, as it has been shown that these effects were suppressed by inhibition of HO-1 activity.^3^,^4^,^15^ Moreover, it has been shown that vascular effects of heme oxygenase and CO are mediated by activation of soluble guanylate cyclase, with increased production of cyclic GMP and activation of potassium channels.^3^,^4^,^15^

Here we found that decreased vascular contractility induced by hemin incubation was associated with a reduction in the relaxation effect of Y-27632. Since Y-27632 is a specific inhibitor of rho-kinase activity,^7^ this effect suggests that following incubation in hemin, the activity of rho-kinase in aortic smooth muscle was partially inhibited. As rho-kinase is involved with calcium sensitivity in vascular smooth muscle, this reduced activity of rho-kinase could explain in part the reduced contractile force developed by aortic rings in response to phenylephrine.

Previously, we have shown that the relaxation effect of Y-27632 was greater in aortic rings from hypertensive rats than in those from normotensive Wistar rats. Moreover, 21 days’ administration of hemin resulted in a reduction in the relaxation effect of Y-27632 on aortic rings from hypertensive rats but was without significant effect on the relaxation of aortic rings induced by Y-27632.^17^ Therefore, although six-hour incubation of aortic rings in hemin resulted in a reduction of the relaxation effect of Y-27632, suggesting a decrease of rho-kinase activity, 21 days’ administration of hemin did not alter the rho-kinase activity in the aortas of Wistar normotensive rat.

However, this long-term administration of hemin decreased the rho-kinase activity in vessels from spontaneously hypertensive rats where this activity was higher than in normotensive rats. To explain the absence of effect on Wistar rat blood pressure during long-term administration of hemin, some mechanisms could have occurred to prevent the reduction of rho-kinase activity and maintain vascular contractility.

## Conclusion

Six-hour incubation of aortic rings in hemin solution resulted in increased heme oxygenase 1 expression and decreased aortic contractility associated with a partial inhibition of rho-kinase activity. The mechanism of this hemin-induced inhibition of rho-kinase activity remains to be elucidated.
